# Mitophagy in Hepatic Insulin Resistance: Therapeutic Potential and Concerns

**DOI:** 10.3389/fphar.2019.01193

**Published:** 2019-10-10

**Authors:** Zuqing Su, Yutong Nie, Xiufang Huang, Ying Zhu, Bing Feng, Lipeng Tang, Guangjuan Zheng

**Affiliations:** ^1^Guangdong Provincial Hospital of Chinese Medicine, the Second Clinical College of Guangzhou University of Chinese Medicine, Guangzhou, China; ^2^The First Affiliated Hospital of Guangzhou University of Chinese Medicine, Guangzhou, China

**Keywords:** hepatic insulin resistance, metabolic syndrome, mitochondrial dysfunction, hepatic fatty acid accumulation, mitophagy

## Abstract

Metabolic syndrome, characterized by central obesity, hypertension, and hyperlipidemia, increases the morbidity and mortality of cardiovascular disease, type 2 diabetes, nonalcoholic fatty liver disease, and other metabolic diseases. It is well known that insulin resistance, especially hepatic insulin resistance, is a risk factor for metabolic syndrome. Current research has shown that hepatic fatty acid accumulation can cause hepatic insulin resistance through increased gluconeogenesis, lipogenesis, chronic inflammation, oxidative stress and endoplasmic reticulum stress, and impaired insulin signal pathway. Mitochondria are the major sites of fatty acid β-oxidation, which is the major degradation mechanism of fatty acids. Mitochondrial dysfunction has been shown to be involved in the development of hepatic fatty acid–induced hepatic insulin resistance. Mitochondrial autophagy (mitophagy), a catabolic process, selectively degrades damaged mitochondria to reverse mitochondrial dysfunction and preserve mitochondrial dynamics and function. Therefore, mitophagy can promote mitochondrial fatty acid oxidation to inhibit hepatic fatty acid accumulation and improve hepatic insulin resistance. Here, we review advances in our understanding of the relationship between mitophagy and hepatic insulin resistance. Additionally, we also highlight the potential value of mitophagy in the treatment of hepatic insulin resistance and metabolic syndrome.

## Introduction

Metabolic syndrome is characterized by hyperglycemia, hyperlipidemia, hypertension, and obesity (Bonamichi et al., 2017; Mohammadbeigi et al., 2018). The increasing prevalence of metabolic syndrome is posing a great threat to human health worldwide (Mohammadbeigi et al., 2018). Insulin resistance is a pathological manifestation that target tissues including liver, muscle, and adipose tissues are less sensitive to the effect of insulin (Barseem and Helwa, 2015). There is a consensus that insulin resistance including liver, skeletal muscle, and adipose tissue insulin resistance is the leading risk factor for metabolic syndrome, obesity, and type 2 diabetes (Bonamichi et al., 2017; Tahrani, 2017). In view of the vital role of liver in glycometabolism and lipid metabolism, hepatic insulin resistance (central insulin resistance) is regarded as the more important risk factor for the development of whole-body insulin resistance and metabolic syndrome (Perseghin, 2009; Ibarra-Reynoso et al., 2014). Therefore, a better understanding of the mechanism by which insulin resistance develops in liver tissue may offer novel therapeutic directions for the treatment or prevention of metabolic syndrome (Ibarra-Reynoso et al., 2014).

Mitochondria are the major sites of fatty acid β-oxidation, which is the major degradation mechanism of fatty acids in hepatocytes and skeletal muscle cells (Chow and From, 2010; Crescenzo et al., 2016). Recent studies have proposed that mitochondrial dysfunction can impair mitochondrial fatty acid β-oxidation, which may cause fatty acid accumulation in liver and skeletal muscle tissues (Chow and From, 2010; Crescenzo et al., 2016). Moreover, accumulating studies have recognized that free fatty acid–induced mitochondrial dysfunction can cause accumulation of hepatic fatty acids, which in turn leads to hepatic insulin resistance (Gonzalez-Franquesa and Patti, 2017; Wu et al., 2018). In the initial study, a decreased number of mitochondria have been found in insulin-resistant skeletal muscle cells, suggesting that mitochondrial function is impaired in insulin-resistant skeletal muscle cells (Perreault et al., 2018). As research progresses, hepatic fatty acid–induced mitochondrial dysfunction has also been proved to play an important role in the development of hepatic insulin resistance (Wang et al., 2017b; Wang et al., 2018; Wang et al., 2019b). Recently, it has been widely recognized that mitochondrial autophagy (mitophagy), a catabolic process, can selectively remove damaged mitochondria by autophagolysosomes to maintain mitochondrial function and energy metabolism (Redmann et al., 2018; Li et al., 2018a). As a mitochondrial quality control mechanism, mitophagy can target and degrade damaged mitochondria to suppress damaged mitochondria-derived reactive oxygen species (ROS), which can dramatically impair healthy mitochondria, leading to mitochondrial dysfunction. Theoretically, mitophagy can preserve mitochondrial function to accelerate fatty acid oxidative degradation and suppress hepatic fatty acid accumulation, which may be conducive to the treatment of hepatic insulin resistance. However, the therapeutic potential and molecular mechanism of mitophagy on hepatic insulin resistance are still unclear.

The purpose of this review is to investigate the complex association between mitophagy and hepatic insulin resistance. First, we review the role and molecular mechanism of insulin-mediated glycometabolism and the associated abnormalities observed in insulin resistance. We then discuss the pivotal role of hepatic insulin resistance in whole-body insulin resistance and metabolic syndrome. Moreover, we discuss the role and molecular mechanism of hepatic fatty acid accumulation on hepatic insulin resistance. Next, we discuss the relationship among mitophagy, mitochondrial dysfunction and hepatic fatty acid accumulation. We also briefly review the related signaling pathways that regulate mitophagy. After discussing the potential role of mitophagy on hepatic insulin resistance with a focus on mitochondrial function and fatty acid oxidation, we put forward a novel idea that mitophagy can preserve mitochondrial function to suppress hepatic fatty acid accumulation, which is conducive to the prevention or treatment of hepatic insulin and metabolic syndrome. Therefore, our major objective is to summarize the role of mitophagy on hepatic insulin resistance and also discuss whether mitophagy is a potential target for the treatment of insulin resistance and metabolic syndrome.

## Insulin Resistance

**Figure 1:**
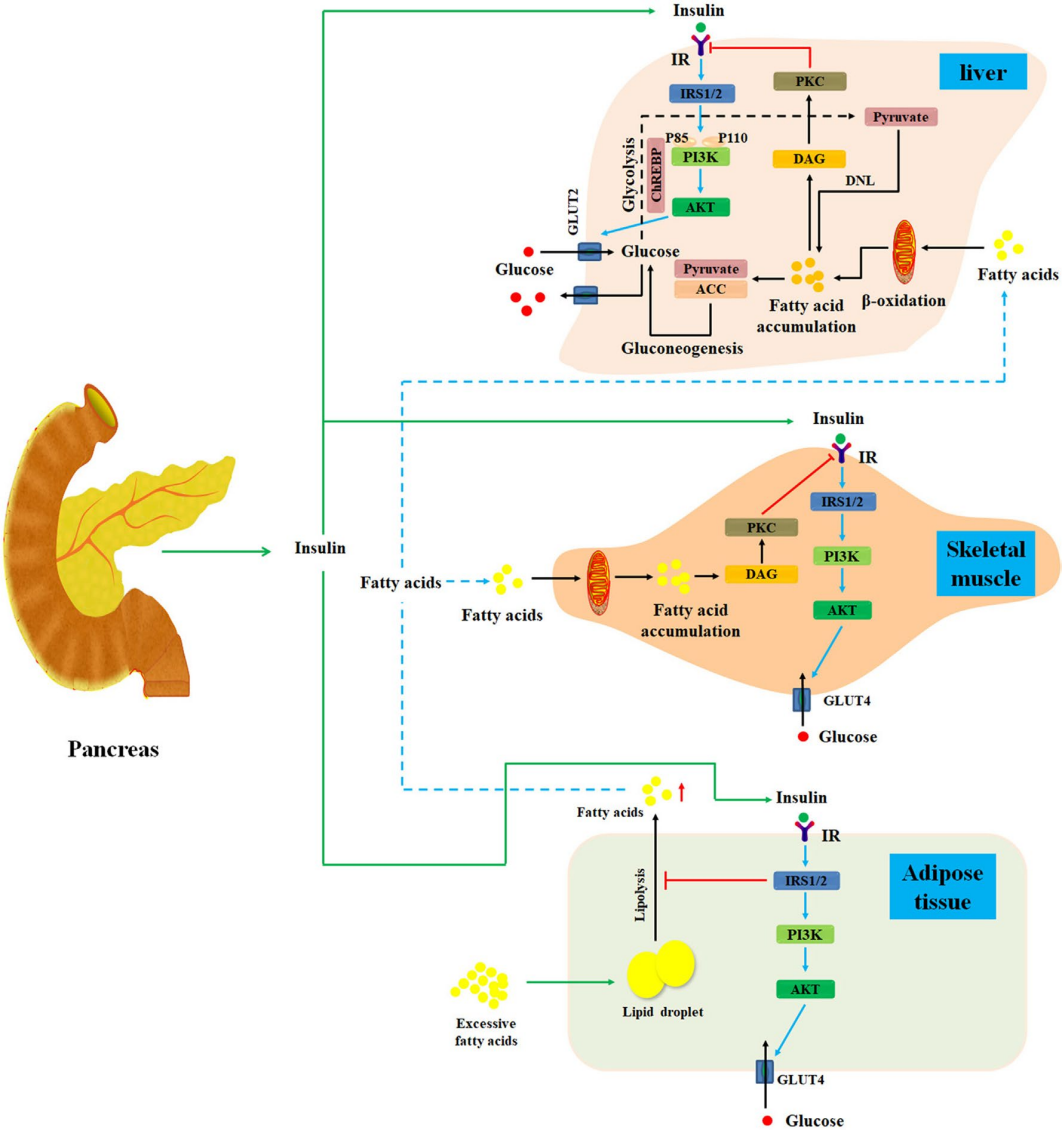
Diagram illustrates the mechanisms of insulin resistance. Normally, after a meal, pancreatic β cells secrete insulin in response to meal-induced increase in blood glucose. First, insulin promotes muscle tissue to assimilate blood glucose and convert it into muscle glycogen and protein. Additionally, insulin also promotes hepatocytes to absorb blood glucose and converts it into liver glycogen. Meanwhile, insulin can inhibit glycogenolysis and gluconeogenesis to reduce postprandial blood glucose in liver tissues. Moreover, insulin can stimulate adipose cells to assimilate blood glucose and convert it into fat. Under starvation conditions, the secretion of insulin will be inhibited, and the hypoglycemic activity of insulin on liver, muscle, and adipose is also suppressed. Under insulin-resistant conditions, lipolysis is promoted in adipose tissue, thereby releasing excess fatty acids into the blood and then transporting them to the liver and skeletal muscle tissue. Then, fatty acids can activate PKC, which may markedly impair insulin signal pathway to cause hepatic and skeletal muscle insulin resistance. IR, insulin resistance; DNL, *de novo* lipogenesis; DAG, diacylglycerol; IRS, insulin receptor substrate; PKC, protein kinase C; ChREBP, carbohydrate responsive element-binding protein; ACC, acetyl-CoA carboxylase; GLUT, glucose transporter.

Insulin, an important endocrine hormone secreted by pancreatic β cells, acts on the insulin receptors (IRs) to regulate the metabolic process of carbohydrate, protein, and lipid in liver, muscle, and adipose tissues (Nicholas et al., 2017; Honka et al., 2018). Under normal circumstances, pancreatic β cells can secrete insulin in response to meal-induced increase in blood glucose (Kang et al., 2017). First, insulin promotes muscle tissue to assimilate blood glucose and convert it into muscle glycogen and protein (Kleinert et al., 2013; Ruby et al., 2017). Second, insulin not only promotes hepatocytes to absorb blood glucose and convert it into liver glycogen, but also inhibits glycogenolysis and gluconeogenesis to reduce postprandial blood glucose (Unger, 2011). Thirdly, insulin stimulates adipose cells to absorb blood glucose and converts it into fat (Gastaldelli, 2011; Sears and Perry, 2015). Collectively, insulin commonly acts on hepatocytes, skeletal muscle, and adipose cells to maintain glycemic homeostasis (**Figure 1**) (Sears and Perry, 2015).

Under insulin-resistant conditions, reduced insulin sensitivity of hepatocytes, skeletal muscle, and adipose cells is found. Insulin resistance is caused by plenty of risk factors such as fatty acid accumulation in liver and skeletal muscle tissues, inflammation, changes in intestinal flora, endoplasmic reticulum stress responses, and environment–gene interactions (Liu et al., 2014; Zhang et al., 2015a; Kikuchi et al., 2018; Vatner et al., 2018; Xu et al., 2018; Crossland et al., 2019). Recently, study has focused on the cause–effect relationship between hepatic fatty acid accumulation and hepatic insulin resistance (He et al., 2013; Finck and Hall, 2015; Dallak, 2018; Vatner et al., 2018).

## Mechanism of Hepatic Insulin Resistance

Liver is the most important organ in the regulation of glycometabolism and lipid metabolism (Ding et al., 2018). Generally, insulin binds to the IR and stimulates the autophosphorylation of IR tyrosine residues, which further activates tyrosine kinase in hepatocytes (Brunetti, 2014; Wang et al., 2015). And then tyrosine kinase phosphorylates insulin receptor substrate 1 and 2 (IRS-1 and IRS-2), which can bind to and activate phosphatidylinositol 3-kinase (PI3K). PI3K then activates the serine/threonine kinase AKT and further increases glucose transporter 2 expression. This process promotes hepatocytes to absorb blood glucose and maintain normal lipid and glucose homeostasis (Cai et al., 2016; Li et al., 2017). Taken together, insulin signal pathway plays a major role in maintaining cell energy metabolism.

Growing evidence has revealed that hepatic lipid accumulation may impair hepatic insulin action (Petersen et al., 2017). Additionally, disordered liver and adipose tissue lipolysis can elevate fatty acid flux to liver and further deteriorate hepatic lipid accumulation, which significantly impair insulin signal pathway and cause hepatic insulin resistance (Yang et al., 2016). In the meantime, the expression of acetyl-CoA carboxylase and pyruvate carboxylase, two specific risk factors for insulin resistance, is also promoted in hepatocytes (**Figure 1**) (Perry et al., 2015; Goedeke et al., 2018). Collectively, hepatic lipid accumulation can cause hepatic insulin resistance through multiple mechanisms (Konstantynowicz et al., 2011). Therefore, it is widely accepted that suppression of hepatic lipid accumulation is a promising approach for the therapy of hepatic insulin resistance (Konstantynowicz et al., 2011; Mohamed and Jornayvaz, 2013; Kubo et al., 2017).

## The Liver Is the First Organ to Develop Insulin Resistance

Accumulating evidence has demonstrated that hepatic insulin resistance is the primary event in free fatty acid–induced whole-body insulin resistance. And muscle and adipose tissue insulin resistance may be the consequence of hepatic insulin resistance (Perry et al., 2014; Badi et al., 2019; Medak and Townsend, 2019). Recent evidence has shown that muscle- and adipose tissue–specific IR knockout mice only show muscle or adipose tissue insulin resistance, but total body glucose metabolism still remains normal (Perseghin, 2009). It is surprising that hepatic IR knockout mice are characterized by liver, muscle, and adipose tissue insulin resistance, as well as fasting and postprandial hyperglycemia (Perseghin, 2009). Further research has confirmed that hepatic lipid accumulation is responsible for hepatic insulin resistance and whole-body insulin resistance (Ye et al., 2016; Sabater et al., 2017; Santos et al., 2017). Furthermore, Lee et al. (2015) have discovered that obese adolescents with fatty liver have a higher risk of systemic insulin resistance than obese adolescents with normal liver. Consistent with previous studies, Rotman and Neuschwander-Tetri (2017) have also proved that hepatic insulin resistance is most likely to be caused by minimal stimulation from hepatic fatty acid accumulation. However, skeletal muscle insulin resistance is less likely to be caused by ectopic fatty acid accumulation in skeletal muscle tissues, and adipose tissue insulin resistance is least likely to be caused by adipose tissue fatty acid accumulation (Rotman and Neuschwander-Tetri, 2017). In summary, growing evidence supports the viewpoint that hepatic fatty acid accumulation can give rise to hepatic insulin resistance and subsequent development of systemic insulin resistance and metabolic disorders.

## What Are Fatty Acids?

Fatty acids are carboxylic acids with a long hydrocarbon chain (Tamil and Nagarajan, 2013). There are various kinds of fatty acids such as essential fatty acid, omega-3 fatty acids, *trans*-fatty acids, and free fatty acids (Williams et al., 2017; Shahidi and Ambigaipalan, 2018).

*Essential fatty acids* mainly consisting of polyunsaturated fatty acids are very important constituents of the human body. It cannot be generated by the human body, while it can only be absorbed from the diet (Amjad et al., 2017).

*Omega-3 fatty acids*, kind of polyunsaturated fatty acids, are found in deep sea fishes, sea dog, and linseed. A growing body of study has shown that omega-3 fatty acids have positive effects on cardiovascular disease, diabetes, cancer, Alzheimer disease, dementia, and depression (Shahidi and Ambigaipalan, 2018).

*Trans-fatty acids*, kind of unsaturated fatty acids, can increase the level of cholesterol and low-density lipoprotein in the blood and have a negative effect on coronary heart disease (Takeuchi and Sugano, 2017; Ahmed et al., 2018).

*Free fatty acids* are by-products of fat metabolism in adipose tissues. The cause–effect relationship between hepatic free fatty acid accumulation and hepatic insulin resistance has been well described (Liu et al., 2015; Kubo et al., 2017).

Given the definite role of free fatty acids on mitochondrial dysfunction and hepatic insulin resistance, this review aims to explore the complex relationship among hepatic fatty acid accumulation, mitochondrial dysfunction, hepatic insulin resistance, and mitophagy (Garcia-Ruiz et al., 2013; Chen et al., 2015; Mohamad et al., 2015).

## The Role of Hepatic Free Fatty Acid Accumulation on Hepatic Insulin Resistance

In general, fatty acids are mainly stored in adipose tissue, and liver tissue is not the physiologic reservoir of fatty acids (Barry and Marry, 2015; Rotman and Neuschwander-Tetri, 2017). Nevertheless, long-term consumption of high-fat diet can lead to excess fatty acids flowing into adipose tissue (Wensaas et al., 2010; Barry and Marry, 2015). When the storage capacity of adipose tissue becomes saturated, superfluous fatty acids will overflow to the blood and further accumulate in nonadipose tissues such as liver, muscle, bone, pancreas, and heart as free fatty acids. This is the process of ectopic fatty acid accumulation (Hagberg et al., 2012; Malinska et al., 2015; Xu et al., 2017). Free fatty acids are the primary sources of hepatic lipid accumulation (Chen et al., 2017c). Under fasting conditions, circulating free fatty acids are the main fuel sources for various tissues except the brain tissue. Hence, plenteous free fatty acids are secreted into the blood by adipose tissue and further transported into the mitochondria of liver and skeletal muscle cells, in which free fatty acids will be oxidized and degraded to provide energy for cellular activity (Grattagliano et al., 2012; Peter and Georg, 2013; Zoladz et al., 2017). Therefore, as one of the important fuel sources, moderate levels of fatty acids in liver and muscle tissues are essential for cellular activities. However, excessive fatty acid accumulation in liver and muscle tissues is a pathological condition (Kubo et al., 2017).

Lipotoxicity is regarded as a pathological state in which high-fat diet impairs the normal metabolism of free fatty acids, resulting in accumulation of free fatty acids in the plasma and tissues (Lee et al., 2018). These fatty acids may impair glucose oxidation and glycogen synthesis and inhibit glucose transport or phosphorylation by suppressing insulin signal pathway and multiple steps of intracellular glucose metabolism (Yazıcı and Sezer, 2017). Therefore, markedly elevated hepatic fatty acids accumulation is a risk factor for hepatic insulin resistance. And the cause–effect relationship between hepatic fatty acid accumulation and hepatic insulin resistance has been confirmed in numerous human and animal researches (Wang et al., 2016; Sabater et al., 2017). The proposed mechanisms of hepatic fatty acid accumulation on hepatic insulin resistance include elevated gluconeogenesis, lipogenesis and chronic inflammation, impaired insulin signal pathway, excessive oxidative stress, and endoplasmic reticulum stress (Pereira et al., 2014; Yoon and Cha, 2014; Wang et al., 2017b).

Hepatic gluconeogenesis, a process of converting nonsugar substances into glucose, is an important regulatory mechanism of blood glucose homeostasis. Hepatic insulin resistance is characterized by overactive gluconeogenesis in liver, which can significantly increase blood glucose level. Glucose-6-phosphatase and glucokinase are two pivotal enzymes that regulate gluconeogenesis (Qi et al., 2018; Song et al., 2018b). Initially, free fatty acids are converted to long-chain fatty acyl coenzyme A by acyl-CoA synthase and then transformed into acetyl CoA in mitochondria of hepatocytes. This will activate hepatic gluconeogenesis to increase hepatic glucose production (Estee and Fiona, 2013; Adeva-Andany et al., 2019). Furthermore, free fatty acids evidently promote the expression of glucose-6-phosphatase to activate gluconeogenesis in liver tissue (Petersen et al., 2017; Tyrrell et al., 2017). Taken together, free fatty acids have a tremendous ability to activate hepatic gluconeogenesis to promote hepatic glucose production, which results in hyperglycemia and hepatic insulin resistance.

*De novo* lipogenesis is another mechanism of free fatty acid–induced hepatic insulin resistance. Free fatty acids promote lipogenic gene expression by regulating transcription factors including sterol regulatory element-binding protein-1c (SREBP-1c), carbohydrate responsive element-binding protein, and peroxisome proliferator-activated receptor γ2 (PPARγ2) (Siculella et al., 2016). Qin et al. (2016) have further identified that free fatty acids increase intracellular lipid accumulation by inhibiting PPARα expression and increasing SREBP-1c level (**Figure 1**).

Moreover, free fatty acid–induced inflammation also plays a central role in the development of hepatic insulin resistance (Wang et al., 2013; Chen et al., 2017c; Zhang et al., 2018). Free fatty acids promote the activity of IKK-β and nuclear factor κB through degrading IκB-α, which further increases the expression of interleukin 1β (IL-1β), tumor necrosis factor α, and IL-6 in liver tissues (Zhang et al., 2015b; Peng et al., 2017). These inflammatory factors can inhibit the activation of PI3K and IRS-1/2 tyrosine phosphorylation to impair insulin signaling pathway (Sharma et al., 2015; Yuan et al., 2016). Additionally, free fatty acids also trigger the expression of hepatic diacylglycerol and protein kinase C-δ (PKCδ) to prevent IRS-1/2 tyrosine phosphorylation, resulting in the occurrence of hepatic insulin resistance (**Figure 1**) (Pereira et al., 2014; Ter Horst et al., 2017).

It is well established that oxidative stress is a crucial risk factor for hepatic insulin resistance. Interestingly, PKCδ, an activator of nicotinamide adenine dinucleotide phosphate (NADPH), can promote the generation of ROS to increase oxidative stress. And ROS in turn promotes the activation of PKCδ, IKK-β, and c-Jun N-terminal kinase (JNK) to exacerbate oxidative stress. In line with previous researches, antioxidants like N-acetyl-l-cysteine and taurine can significantly prevent hepatic insulin resistance through suppression of excessive oxidative stress in rats (Guo-Guang et al., 2013; Pereira et al., 2014; Pereira et al., 2015; Cui et al., 2017; Villagarcía et al., 2018). Moreover, Pereira et al. (2014) have reported that hepatic fatty acid accumulation may activate PKCδ to increase NADPH oxidase-dependent oxidative stress, which further promotes the expression of IKK-β/JNK to impair hepatic insulin signaling pathway and finally cause hepatic insulin resistance. In summary, the pathway of free fatty acid–induced hepatic insulin resistance is free fatty acids → PKCδ → NADPH oxidase → oxidative stress → IKK-β/JNK → insulin signaling pathway → hepatic insulin resistance (Pereira et al., 2014).

## Mitophagy and Lipid Metabolism

Autophagy widely existing in eukaryotic cells is essential for maintaining cellular energy homeostasis (Vargas et al., 2017). It is a conservative self-digestion process that relies on lysosomes, by which excessive fatty acids, damaged cell structures, and organelles can be degraded by lysosomal enzymes (Miyamoto and Heller, 2016; Chu et al., 2018). Depending on how they bind to lysosome, autophagy can be divided into macroautophagy, microautophagy, and chaperone-mediated autophagy (Cerri and Blandini, 2018). Moreover, autophagy can be also classified as selective autophagy and nonselective autophagy (Kim et al., 2016). Mitophagy is a highly selective autophagy, which can selectively degrade damaged mitochondria through macroautophagy (McWilliams et al., 2018). To be more specific, damaged mitochondria are swallowed up by LC3-positive autophagosomes, which subsequently fuse with lysosomes and degrade these mitochondria (McWilliams et al., 2018). Recently, a growing body of research has shown that mitophagy is of vital importance for mitochondrial quality control and mitochondrial dynamics including biosynthesis and degradation (Youle and Narendra, 2011; Michael et al., 2015; Kim et al., 2016).

There is a consensus that mitochondrial dysfunction is closely involved in the development of Parkinson disease and Alzheimer disease (Ganguly et al., 2017). Fortunately, these diseases can be reversed by mitophagy (Kerr et al., 2017; Sliter et al., 2018). Recent research has shown that impaired mitophagy is responsible for the development of insulin resistance diseases, such as metabolic syndrome, type 2 diabetes, obesity, and hyperlipidemia (Seillier et al., 2015; Rovira-Llopis et al., 2017; Che et al., 2018). Nevertheless, whether mitophagy can improve insulin resistance diseases is still not clear (Gonzalez-Franquesa and Patti, 2017). Therefore, we then explore the potential role and molecular mechanism of mitophagy on insulin resistance with a focus on lipid metabolism. We hope to elucidate the connection between mitophagy and insulin resistance and finally reveal a novel therapeutic target for insulin resistance.

## Signaling Pathways Involved in Mitophagy

Mitophagy selectively degrades damaged mitochondria, which contributes to mitochondrial quality control and maintenance of mitochondrial function. There are three main types of mitophagy: PINK1/Parkin-mediated mitophagy, BNIP3/NIX-mediated mitophagy, and FUN14 domain-containing 1 (FUNDC1)–mediated mitophagy (Sato and Furuya, 2017; Yuan et al., 2017; Li et al., 2018c).

### PINK1/Parkin-Mediated Mitophagy

It is well recognized that the mitochondrial serine/threonine kinase PTEN-induced putative kinase 1 (PINK1) and the E3 ubiquitin ligase Parkin are two important proteins mediating mitophagy in mammalian cells (Williams et al., 2015; Harper et al., 2018). In healthy mitochondria, PINK1 is transported into the inner mitochondrial membrane and then cleaved by the inner membrane protease PARL. Ultimately, the truncated form of PINK1 is released into the cytoplasm for N-terminal recognition and then degraded by the proteasome to remain at a low basal level (Wu et al., 2015; Wang et al., 2019a). In damaged mitochondria, mitochondrial membrane potential (澔ΔΨm) is insufficient to transport PINK1 to the inner mitochondrial membrane. Therefore, PINK1 mainly locates on the outer mitochondrial membrane of depolarized mitochondria and then phosphorylates some outer mitochondrial membrane protein (Greene et al., 2012; Lemasters and Zhong, 2018). This process can recruit autophagy adaptors including NDP52 and OPTN, which can bind to the double membrane vacuoles (autophagosomes). Therefore, damaged mitochondria can be captured by autophagosomes, which then fuse with lysosomes to degrade these mitochondria (Michael et al., 2015; Moreira et al., 2017). In order to further activate mitophagy, PINK1 located on the outer mitochondrial membrane of damaged mitochondria recruits cytosolic Parkin to damaged mitochondria to activate mitophagy by Mnf1 and Mnf2 phosphorylation. Additionally, PINK1 can phosphorylate Ser65 in ubiquitin and ubiquitin-like domain of Parkin to enhance Parkin E3 ubiquitin ligase activity to induce mitophagy (Wang et al., 2019a).

Generally, Parkin promotes mitophagy through two main pathways: First, Parkin ubiquitinates mitochondrial GTPase such as Miro and Mitofusin proteins including Mnf1 and Mnf2 to cause mitochondrial fragmentation and motility arrest, followed by sequestration and degradation of damaged mitochondria by autophagolysosomes (Eid et al., 2016; Moreira et al., 2017). Additionally, Parkin induces the ubiquitination of mitochondrial outer membrane proteins such as voltage-dependent anion channel, which can be identified by ubiquitin-binding adaptors including histone deacetylase 6 and SQSTM1/p62. This process contributes to propelling damaged mitochondria to the autophagic isolation membrane, for subsequent degradation of damaged mitochondria by mitophagy (Moreira et al., 2017).

Recent study has found that hepatic fatty acid accumulation can cause damaged mitochondria accumulation, which can impair mitochondrial respiratory chain function and fatty acid oxidative degradation (Ashrafi and Schwarz, 2013; Wu et al., 2015). Surprisingly, PINK1/Parkin-mediated mitophagy can reverse mitochondrial dysfunction and preserve mitochondrial function through eliminating damaged mitochondria in time (Wu et al., 2015; Nguyen et al., 2016; Xiong et al., 2018; Song et al., 2018a; Wang et al., 2019a).

### BNIP3/NIX -Mediated Mitophagy

In response to hypoxia and nutrient deprivation, programmatic elimination of mitochondria by mitophagy is induced to inhibit excessive mitochondrial mass and maintain mitochondrial function. This kind of stress-responsive mitophagy is regulated by two key mitophagy adaptors: BCL-2/adenovirus E1B interacting protein 3 (BNIP3) and Nip-like protein X (NIX). BNIP3 and NIX, two pivotal BCL-2 homology domain 3–only proteins, play important roles in mitophagy–mediated mitochondrial quality control (Lampert et al., 2019; Xu et al., 2019). It is noteworthy that PINK1 and Parkin activate mitophagy through indirect binding to autophagosomes, while BNIP3 and NIX activate mitophagy through direct binding to autophagosomes (Moreira et al., 2017).

Initially, BNIP3/NIX may promote the moderate expression of ROS to enhance mitophagy (Scherz-Shouval and Elazar, 2011). Second, phosphorylation is an important process in BNIP3-induced mitophagy. The phosphorylation can promote BNIP3 to bind to LC3 II, a molecule critical for autophagosome formation, and phosphorylation of Ser24 on BNIP3 can further promote the affinity (Moreira et al., 2017). Additionally, Beclin-1, the mammalian ortholog of yeast Atg6, activates autophagy in the form of Beclin-1–Vps34 (lipid kinase Vps-34 protein)–Vps15 (lipid kinase Vps-15 protein) complexes (Ma et al., 2014; Wang et al., 2017a). Bcl-2 and Bcl-XL can bind to the BH3 domain of Beclin-1 in the form of Beclin-1–Bcl-2 and Beclin-1–Bcl-XL complexes to inhibit mitophagy. However, BNIP3 and NIX can compete with Beclin-1 to bind to Bcl-2 and Bcl-XL. And then Beclin-1 is liberated from these complexes to activate mitophagy (Chiara Maiuri et al., 2014; Chiang et al., 2018). Thirdly, Ras homolog enriched in brain (Rheb) can suppress mitophagy through activating mammalian target of rapamycin (mTOR), while BNIP3 can block Rheb-mTOR signaling pathway to enhance mitophagy (Lin et al., 2014; Gong et al., 2017). Moreover, NIX can directly bind to LC3. LC3 can bind to γ-aminobutyric acid receptor–associated protein (GABARAP) to form LC3–GABARAP complex, which promotes the mobilization of autophagosomes to damaged mitochondria (Moreira et al., 2017).

Taken together, metabolic stress, including hypoxia, nutrient deprivation, and fatty acid–induced dysfunctional mitochondria accumulation, can induce BNIP3/NIX-mediated mitophagy to clear damaged mitochondria and preserve mitochondrial integrity and function (Danielle et al., 2012; Moreira et al., 2017).

### FUNDC1-Mediated Mitophagy

FUN14 domain-containing 1 located on the mitochondrial outer membrane contains a motif of Y (18) xxL (21), an LC3-interacting region (LIR), at the N-terminal (Lei et al., 2012). Under hypoxic conditions, similar to BNIP3 and NIX, FUNDC1 can directly bind to LC3 through its LIR motif to induce mitophagy (Wenxian et al., 2014; Yu et al., 2019a). Under nonstress conditions, Sc and CK2 kinases can phosphorylate Tyr-18 in LIR motif of FUNDC1 to interfere with the interaction between FUNDC1 and LC3, which subsequently impairs FUNDC1-mediated mitophagy (Wenxian et al., 2014). Additionally, FUNDC1 regulates mitophagy-mediated mitochondrial quality control through interacting with fission and fusion machinery components. For example, under hypoxic conditions, phosphoglycerate mutase 5, a mitochondrial Ser/Thr protein phosphatase, can dephosphorylate FUNDC1 and subsequently impair the interaction of FUNDC1 with mitochondrial fusion protein OPA1 to suppress mitochondrial fusion. Under normoxic conditions, bits of FUNDC1 have been identified in the endoplasmic reticulum–mitochondria contact sites. Surprisingly, in response to hypoxic stress, FUNDC1 substantially interacts with the endoplasmic reticulum resident protein calnexin and further recruits mitochondrial fission protein DRP1 to activate mitochondrial fission (Palikaras et al., 2018). Moreover, under hypoxic conditions, ULK1 (UNC-51 like kinase 1), a pivotal component of autophagy initiation complex, can phosphorylate Ser-17 in LIR motif of FUNDC1 to promote the interaction between FUNDC1 and LC3 to induce mitophagy (Springer and Macleod, 2016). Similar to FUNDC1, hypoxia-induced mitophagy is also regulated by BNIP3 and NIX. Under hypoxic conditions, hypoxia inducible factor 1 can increase the expression of BNIP3 and NIX to enhance mitophagy (Palikaras et al., 2018). Notwithstanding the crosstalk among FUNDC1, BNIP3, and NIX is still confusing, their coordinated action is pivotal for mitophagy-based mitochondrial quality control.

Recent study has shown that BNIP3-, NIX-, and FUNDC1-mediated mitophagy plays a critical role in the treatment of lipid metabolism, hepatocellular carcinoma, hepatic insulin resistance, alcoholic liver disease, hepatic steatosis, and liver injury (Danielle et al., 2012; Williams and Ding, 2015; Chao et al., 2018; Liu et al., 2018; Li et al., 2019; Yu et al., 2019b). Nevertheless, research of mitophagy on liver diseases is still in its infancy, and its role and molecular mechanism involved in liver diseases are still needed to be confirmed by extensive experiments. Given the central role of mitochondrial quality control on energy homeostasis and adaptive response, pursuit of the role and molecular mechanism of mitophagy on liver diseases should be a field full of surprises, which may provide a novel perspective for the prevention and treatment of insulin resistance and metabolic syndrome.

## Mitophagy Prevents Hepatic Fatty Acid Accumulation

Mitochondrial fatty acid oxidation is an important physiological process for fatty acid degradation and ATP production. Human body can absorb abundant fatty acids from diets and store them in adipose tissue. In the fasting state, these fatty acids are released from adipose tissue and further transported into the mitochondria of hepatocytes and skeletal muscle cells, in which fatty acids are oxidized for ATP production (Zhang et al., 2012). Therefore, normal mitochondrial function plays a pivotal role in regulating lipid metabolism and cellular energy supply. It is worth noting that healthy mitochondria can suppress fatty acid accumulation in liver and muscle tissue through accelerating fatty acid β-oxidation (Rambold et al., 2015; Sharma et al., 2018).

Accumulating evidence has shown that defective hepatic mitochondrial respiration characterized by damaged mitochondria accumulation can impair mitochondrial fatty acid β-oxidation, which sequentially causes various adverse consequences, such as excessive ROS, reduced ATP production, and hepatic fatty acid accumulation (Serviddio et al., 2010; Chistiakov et al., 2014; Crescenzo et al., 2016). There is a consensus that mitophagy, one of the prominent approaches for mitochondrial quality control, can remove damaged mitochondria to restore mitochondrial quality and mitochondrial function (Chakraborty et al., 2018; Saxena et al., 2019). The positive role of mitophagy on the scavenging of ectopic fatty acid accumulation has been clearly demonstrated. In an animal model of alcoholic fatty liver, Eid et al. (2016) have demonstrated that Parkin-mediated mitophagy can selectively clear damaged mitochondria to maintain mitochondrial quality, which is pivotal to the inhibition of hepatic lipid accumulation. In line with Eid and colleagues’ standpoint, Williams et al. (2015) have found that Parkin knockout mice are more susceptible to alcohol-induced liver steatosis than wild-type mice. They have speculated that Parkin-mediated mitophagy plays a pivotal role in maintaining mitochondrial function, which can accelerate fatty acid oxidation and sequentially suppress fatty acid accumulation in liver tissue (Williams et al., 2015). In an animal model of high-fat diet–induced nonalcoholic fatty liver, the results have indicated that impaired PINK1/Parkin-dependent mitophagy may be responsible for hepatic fatty acid accumulation (Liu et al., 2018). As expected, quercetin, a common flavonoid, can activate PINK1/Parkin-mediated mitophagy to accelerate mitochondrial fatty acid oxidation and inhibit hepatic fatty acid accumulation in an animal model of high-fat diet–induced nonalcoholic fatty liver. Additionally, quercetin also activates PINK1/Parkin-dependent mitophagy to prevent oleic acid/palmitic acid–induced lipid accumulation in HepG2 cells (Liu et al., 2018). Moreover, linseed oil, exenatide, melatonin, akebia saponin D, and sirtuin 3 have also been shown to suppress hepatic lipid accumulation through activating mitophagy (**Table 1**).

**Table 1:** The role and molecular mechanisms of natural or synthesized compounds-induced mitophagy on liver diseases.

Compound	Disease model	Mechanism	Protein	Reference
Quercetin	Mice with nonalcoholic fatty liver disease (NAFLD) and free fatty acid–treated HepG2 cells	Activating mitophagy to improve hepatic steatosis	PINK1 ↑, Parkin ↑, Beclin-1↑, LC3-II/I ↑, and p62 ↓	(Liu et al., 2018)
Linseed oil	Obese mice	Activating mitophagy to improve hepatic insulin resistance, hepatic mitochondrial biogenesis, hepatic lipid accumulation	Parkin ↑, FUNDC1 ↑, LC3-II/I ↑, and p62 ↓	(Yu et al., 2019b)
Exenatide	Mice with NAFLD	Activating mitophagy to reduce oxidative stress and NLRP3 inflammasome in liver tissue	LC3-II/I ↑, Beclin-1 ↑, Parkin ↑, BNIP3 ↑, NLRP3↓, and IL-1β ↓	(Shao et al., 2018)
Melatonin	Mice with NAFLD and palmitic acid (PA)–treated primary hepatocytes	Promoting Drp1-mediated mitochondrial fission and BNIP3-dependent mitophagy to rescues mitochondrial respiratory function	Drp1 ↑, Atg5 ↑, Beclin-1 ↑, mito-LC3II ↑, and BNIP3 ↑	(Zhou et al., 2018)
Akebia saponin D	Oleic acid–treated BRL cells	Alleviating hepatic steatosis through promoting BNIP3-mediated mitophagy	mTOR ↓, LC3II ↑, and BNIP3 ↑	(Gong et al., 2018)
Sirtuin 3	Mice with NAFLD and PA -treated primary hepatocytes	Alleviating hepatic steatosis through promoting BNIP3-mediated mitophagy	Mito-LC3II ↑, Atg5 ↑, Beclin-1 ↑, and BNIP3 ↑.	(Li et al., 2018b)

Moreover, recent study has shown that macrophage can infiltrate into white adipose tissue to promote lipolysis and then release fatty acids into the bloodstream. These fatty acids may be transported to liver tissue and then promote lipid synthesis by esterification. Fatty acids can promote the activation of PKC to impair insulin signal pathway, subsequently causing hepatic insulin resistance (Samuel and Shulman, 2016). Wu et al. (2019) have found that impaired mitophagy can accelerate macrophage infiltration in white adipose tissue in high-fat diet–fed mice with FUNDC1 knockout. They have also demonstrated that impaired mitophagy can promote macrophage infiltration to activate MAPK signal pathway and inflammatory response to impair mitochondrial quality control, subsequently causing insulin resistance and hepatic steatosis (Wu et al., 2019). Collectively, these evidences have suggested that mitophagy can suppress macrophage-induced inflammatory response and improve mitochondrial quality control to suppress hepatic insulin resistance and steatosis.

In a word, mitophagy can prevent hepatic fatty acid accumulation *via* maintaining mitochondrial function and accelerating mitochondrial fatty acid oxidation. However, the study of mitophagy on liver diseases is still in its infancy, and its role and molecular mechanism are also still needed to be confirmed by extensive experiments.

## Mitophagy as Therapeutic Intervention in Hepatic Insulin Resistance

Mitochondrion is the major site of aerobic respiration and the main energy production center. Glucose, fatty acids and amino acids are substrates for mitochondrial energy production. Mitochondria play central roles in substrate oxidation, tricarboxylic acid cycle, oxidative phosphorylation, cell proliferation, cell metabolism, and programmed cell death (Montgomery and Turner, 2015). There are plenteous methods for the determination of mitochondrial function, including the protein and mRNA expressions of mitochondrial-encoded genes CYTB and COX1; respiratory chain complexes I, II, and III; nuclear-encoded genes PGC1α; mitochondrial enzyme activity; mitochondrial size and shape; mitochondrial quantity; and ROS production (Montgomery and Turner, 2015; O’brien et al., 2017; Trotta and Chipuk, 2017; Lima et al., 2018; Lv et al., 2019). However, there is no recognized method available for the detection of mitochondrial respiratory chain function. Therefore, it is urgent to develop a recognized method for mitochondrial respiratory chain function evaluation.

Given the central role of mitochondria in cellular energy metabolism, mitochondrial dysfunction is predominantly referred to as defective mitochondrial oxidative phosphorylation, which is characterized by impaired substrate oxidation and damaged mitochondria accumulation (Crescenzo et al., 2016). In general, damaged mitochondria accumulation can cause excessive ROS production, which will destroy mitochondrial oxidative phosphorylation and substrate oxidation, thereby inhibiting fatty acid oxidative degradation and accelerating fatty acid accumulation (Finck and Hall, 2015; Crescenzo et al., 2016; Horst et al., 2017). As a result, fatty acids especially diacylglycerol and ceramide may excessively accumulate in liver tissue. Previous report has shown that diacylglycerol can activate PKC to impair insulin signal pathway (Ditte et al., 2016; Ter Horst et al., 2017). Moreover, ceramide also activates PKCζ to suppress protein kinase AKT and further inhibits insulin signal pathway (Montgomery and Turner, 2015; Sajan et al., 2015; Chen et al., 2017b). In conclusion, mitochondrial dysfunction gives rise to hepatic fatty acid accumulation, which ultimately impairs insulin signal pathway and causes hepatic insulin resistance.

Insulin plays a central role in lipid and glucose metabolism and also promotes mitochondrial function characterized by enhanced mitochondrial oxidative metabolism and ATP production in hepatocytes (Kim et al., 2015; Ruegsegger et al., 2019). However, insulin resistance also leads to mitochondrial dysfunction. Previous evidence has demonstrated that hepatic insulin resistance exacerbates lipid deposition, oxidative stress, lipid peroxidation, and mitochondrial dysfunction in liver tissues (Goodpaster, 2013). Therefore, a vicious circle between dysfunctional mitochondrial respiration and hepatic insulin resistance is established.

Growing evidence has shown that mitophagy may be a promising therapeutic target for hepatic insulin resistance. Marycz et al. (2018) have speculated that mitophagy is a repair mechanism responsible for cellular energy homeostasis and cell survival in insulin resistance hepatocytes and adipose cells. Qi et al. (2016) have also found that impaired mitophagy dramatically exacerbates high-fat diet–induced insulin resistance. Fortunately, enhanced mitophagy remarkably protects mice from high-fat diet–induced insulin resistance. Therefore, they consider that mitophagy should be considered as a protective response to high-fat diet–induced insulin resistance (Qi et al., 2016).

Currently, mitochondrial dysfunction is recognized as a pivotal risk factor for insulin resistance and metabolic syndrome (Crescenzo et al., 2016; Sarparanta et al., 2017). Autophagy is responsible for eliminating misfolded proteins and dysfunctional organelles such as aged or dysfunctional mitochondria and endoplasmic reticulum (Che et al., 2018). Therefore, as a selective autophagy, impaired mitophagy may fail to eliminate damaged mitochondria in time, which further causes excessive accumulation of damaged mitochondria in hepatocytes and triggers mitochondrial dysfunction (Crescenzo et al., 2016). Therefore, researchers have suggested two therapeutic strategies for improving mitochondrial dysfunction: One is to increase the quantity of mitochondria by enhancing its biosynthesis. The other is to remove damaged mitochondria by mitophagy. Considering that clearance of dysfunctional mitochondria is more important for mitochondrial homeostasis including mitochondrial function and biosynthesis, more attention has been focused on the removal mechanism of dysfunctional mitochondria in insulin-resistant conditions (Gonzalez-Franquesa and Patti, 2017; Kalavalapalli et al., 2018; Marycz et al., 2018). There is no doubt that mitophagy-mediated removal of dysfunctional mitochondria should be the center of attention. Growing evidence has also shown that mitophagy may be a potential therapeutic target for hepatic insulin resistance (Mottillo et al., 2016; Zhang et al., 2017). Very recently, a limited number of researches have reported that mitophagy may improve mitochondrial quality and accelerate fatty acid oxidative degradation to suppress hepatic insulin resistance and lipid accumulation. However, the cause–effect relationship between hepatic insulin resistance and mitophagy is also still needed to be confirmed by substantial experiments (Pickrell et al., 2013; Chen et al., 2017a).

## Conclusion

**Figure 2:**
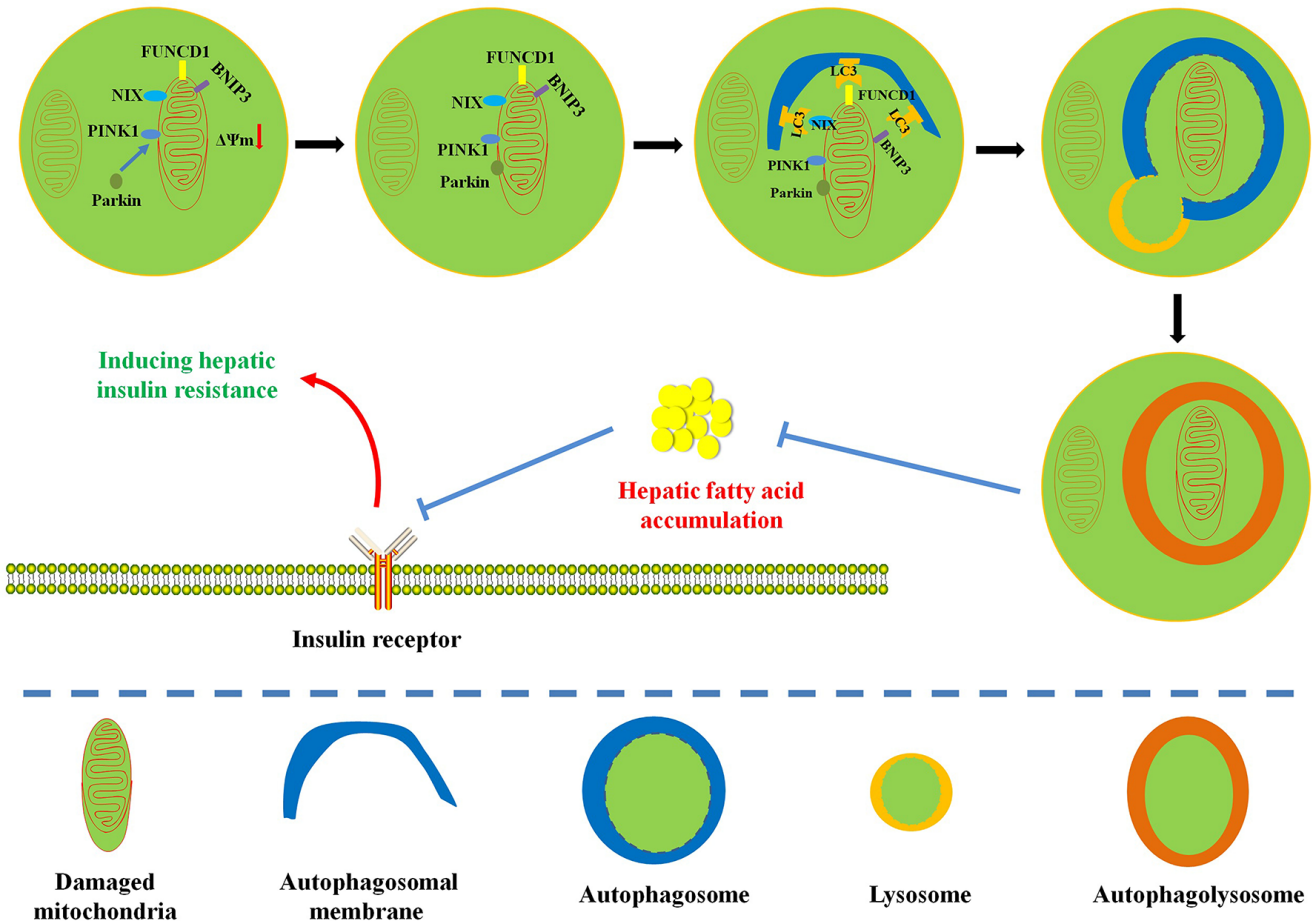
Diagram illustrates the possible protective mechanism of mitophagy on hepatic fatty acid–induced hepatic insulin resistance. Enhanced mitophagy can degrade damaged mitochondria to restore mitochondrial function and accelerate mitochondrial fatty acid oxidation, which is beneficial to the reversion of hepatic fatty acid accumulation and the improvement of hepatic insulin resistance.

This review focuses on the complex relationship among hepatic insulin resistance, hepatic fatty acid accumulation, mitochondrial dysfunction, and mitophagy. Mitochondria play pivotal roles in the regulation of fatty acid metabolism. Extensive studies have indicated that mitochondrial dysfunction plays a central role in hepatic fatty acid–induced hepatic insulin resistance (Montgomery and Turner, 2015; Wang et al., 2017b). Interestingly, mitophagy contributes to mitochondrial quality control and the maintenance of mitochondrial function by selectively degrading defective mitochondria. At present, the protective mechanism of mitophagy on hepatic insulin resistance still needs further experimental confirmation. In this review, we summarize some evidences: (1) Influx of fatty acids into liver tissue can cause mitochondrial dysfunction; (2) mitochondrial dysfunction can further obstruct the timely elimination of fatty acids and induce hepatic fatty acid accumulation; (3) hepatic fatty acid accumulation is responsible for the pathogenesis of lipotoxicity and inflammation; (4) lipotoxicity and inflammation can interfere with insulin signaling pathway to cause hepatic insulin resistance; (5) interestingly, enhanced mitophagy can selectively remove damaged mitochondria to improve mitochondrial dysfunction and restore mitochondrial function, which is beneficial to the elimination of hepatic fatty acids; (6) mitophagy-mediated recovery of mitochondrial function can reverse hepatic fatty acid accumulation–induced hepatic insulin resistance. The association between mitophagy and hepatic insulin resistance is still not completely understood, in part due to various methods for detecting mitochondrial function (Chow and From, 2010; Montgomery and Turner, 2015). Therefore, given the confirmed relationship between hepatic fatty acid–induced mitochondrial dysfunction and hepatic insulin resistance, we hypothesize that mitophagy can remove damaged mitochondria to restore mitochondrial function and promote the oxidative degradation of fatty acids (**Figure 2**). This physiological process is conducive to the inhibition of hepatic fatty acid accumulation and hepatic insulin resistance (**Figure 2**). A limited number of studies have proved that enhanced mitophagy can inhibit hepatic lipid accumulation to improve hepatic insulin resistance. However, the definite relationship between mitophagy and hepatic insulin resistance still needs further experimental confirmation (Yang et al., 2014; Seillier et al., 2015; Corsa et al., 2019). Moreover, energy metabolism and organismal homeostasis attribute to tight coordination between mitochondrial biogenesis and degradation. Certainly, pursuit of natural or synthesized compounds possessing mitochondrial biogenic and mitophagic activities may provide novel insights into the therapeutic interventions for mitochondria-related diseases such as hepatic insulin resistance and metabolic syndrome.

## Author Contributions

ZS, YN, and XH drafted the manuscript. ZS, XH, YZ, BF, and LT created the figures and performed literature searches. ZS and GZ revised the manuscript and edited the final draft.

## Funding

This work was supported by grants from the National Natural Science Foundation of China (grant 81703770); the Science and Technology Planning Project of Guangdong Province, China (grant 2014A020221080 and 2016A020226048); the Administration of Traditional Chinese Medicine of Guangdong Province, China (grant 20172059); the Chinese Medicine Scientific Research and Technology Research Projects of Guangdong Provincial Hospital of Chinese Medicine (grant YN2018QJ04 and YN2019QJ10); and Guangdong Provincial Key Laboratory of Chinese Medicine for Prevention and Treatment of Refractory Chronic Diseases (grant 2018B030322012).

## Conflict of Interest

The authors declare that the research was conducted in the absence of any commercial or financial relationships that could be construed as a potential conflict of interest.
